# Fluorescence‐Based Multiplex Western Blot to Simultaneously Detect the Insulin‐Like Growth Factor‐1 (IGF‐1) Isoforms

**DOI:** 10.1002/elps.8116

**Published:** 2025-03-19

**Authors:** Matteo Bocconcelli, Fabiana Fanelli, Roberta Saltarelli, Mauro De Santi, Rita Barone, Elena Barbieri, Giosuè Annibalini

**Affiliations:** ^1^ Department of Biomolecular Sciences University of Urbino Carlo Bo Urbino Italy; ^2^ Child Neurology and Psychiatry Unit, Department of Clinical and Experimental Medicine University of Catania Catania Italy; ^3^ Research Unit of Rare Diseases and Neurodevelopmental Disorders Oasi Research Institute‐IRCCS Troina Italy

**Keywords:** insulin‐like growth factor‐1, isoforms, multiplex fluorescent Western blotting, prohormones

## Abstract

Insulin‐like growth factor‐1 (IGF‐1) is critical for tissue growth and development. The *IGF‐1* gene contains six exons and due to alternative splicing three different isoforms might be produced: the IGF‐1Ea, Eb, and Ec prohormones (proIGF‐1s). These proIGF‐1s share the same IGF‐1 mature sequence, which is responsible for the IGF‐1 receptor binding but differ in their carboxy‐terminal extensions called Ea‐, Eb‐, and Ec‐peptides. Several lines of evidence indicate that E‐peptides control the intracellular proIGF‐1s localization and maturation. Here, we present a multiplex Western blotting system able to simultaneously discriminate and quantify mature IGF‐1, proIGF‐1s and E‐peptides within the same sample. HEK293 cells were transiently transfected with plasmids containing the *IGF‐1Ea*, *IGF‐1Eb*, or *IGF‐1Ec* isoform or an empty vector. Two different primary antibodies, which recognize the mature sequence or the common region of E‐peptides, were used to detect IGF‐1 isoforms, which were subsequently distinguished with secondary antibodies conjugated to different fluorophores. Our results demonstrate the feasibility of simultaneously detecting different IGF‐1 isoforms using two primary antibodies directed against different epitopes of proIGF‐1s, combined with fluorescence‐conjugated secondary antibodies. Furthermore, this dual‐epitope strategy increases the specificity of protein detection, making it a valuable tool for studying the diverse roles of IGF‐1 isoforms in biological processes.

**Abbreviations**
HEK293 cells: human embryonic kidney cells
IGF‐1: insulin‐like growth factor‐1

## 1 Introduction

The Western blotting (WB) technique is one of the most widely used and informative methods in biological research. It is extensively employed to determine the presence, size and relative abundance of specific proteins in complex mixtures. WB is normally performed by separating proteins using gel electrophoresis, transferring proteins to a nitrocellulose or polyvinylidene difluoride (PVDF) membrane, labeling the target protein with a specific antibody followed by visualization of the antibody binding. The visualization methodology in WB has evolved over time to improve both safety and sensitivity and is typically attained through a secondary antibody conjugated to an enzyme or a fluorescent molecule [1, 2, 3, 4]. The chemiluminescence approach is based on an enzymatic reaction between hydrogen peroxide and luminol. The reaction is catalyzed by antibody‐conjugated horseradish peroxidase (HRP) and results in light emission. Traditional chemiluminescence detection emits light at a relatively low intensity and for a short period of time. Enhanced chemiluminescence detection was developed to amplify the signal and has become the dominant technique for revealing proteins in WB [5]. This technique enables the detection of picograms of protein usually visualized with x‐ray film or a digital charge‐coupled device (CCD) imager. Despite its sensitivity, chemiluminescence‐based WB is a one‐color method that detects a single protein target and does not facilitate normalization or comparative analysis. In recent years, the use of fluorescent probes in WB stands out for their convenience, safety, sensitivity, and straightforward quantification [6, 7, 8, 9, 10]. The main advantage of using fluorescently labeled antibody probes rather than chemiluminescence is the ability to multiplex analysis such as the visualization of different target proteins with similar molecular weight (MW) such as phosphorylated and non‐phosphorylated isoforms of a protein. Furthermore, another advantage is the increased linearity and accuracy of quantification since no enzymatic amplification is involved and blots can be catalogued for extended periods of time when stored properly. The main drawbacks of fluorescent WB are: less sensitive than chemiluminescence, and strong high background fluorescence of membranes. In this regard, the introduction of antibodies directly labelled with near‐infrared (IR) dyes, the use of low fluorescence membrane and new fluorescence‐optimized blocking buffer have significantly improved fluorescent WB sensitivity [11, 12]. Furthermore, fluorescent WB often uses less toxic reagents but is typically more expensive due to the high cost of fluorophore‐conjugated antibodies and specialized imaging equipment. In contrast, chemiluminescence WB is generally more cost‐effective for protein detection but may raise toxicity concerns due to the use of chemicals such as luminol and signal enhancers. In this paper, we present a fluorescent‐based WB able to reveal different protein isoforms of human insulin‐like growth factor‐1 (IGF‐1). IGF‐1 is a growth factor critical for tissue development and implicated in the onset and progression of various malignancies [13]. Alternative splicing of the *IGF‐1* gene produces three different mRNA isoforms in humans: *IGF‐1Ea*, *IGF‐1Eb*, and *IGF‐1Ec* [14, 15]. The translation of these mRNA variants gives rise to three different IGF‐1 prohormones (proIGF‐1s) in humans: proIGF1Ea, proIGF1Eb and proIGF1Ec [16]. These prohormones contain the same central region, named mature IGF‐1 peptide or core region, and three C‐terminal domains called Ea‐, Eb‐, and Ec‐peptides or domains. The human Ea‐domain is composed of 35 amino acids (aa); the first 16 aa of Ea‐domain are common in all E‐domains, while 19 aa are unique to this isoform. The human Ea‐domain contains a *N*‐glycosylation site on the asparagine residue at position 92 (N92) [17]. Accordingly, both unglycosylated proIGF‐1Ea (11.7 kDa) and glycosylated proIGF‐1Ea (∼17–22 kDa) were found in normal and IGF‐1‐overexpressing cells [17, 18]. The human Eb‐ and Ec‐domains contain the 16 common aa and 61 and 24 additional isoform‐specific aa, respectively, with a predicted MW of 16.5 kDa for proIGF‐1Eb and 12.4 kDa for proIGF‐1Ec. The human Eb‐ and Ec‐domains lack potential *N*‐linked glycosylation consensus sequences [17]. Several steps are required for the formation and secretion of the mature IGF‐1 peptide. Initially, the *IGF‐1* mRNA isoforms are translated as pre‐prohormones containing a signal peptide, the core region, and the Ea‐, Eb‐, or Ec‐peptides. Following cleavage of the signal peptide, proIGF‐1s undergo additional posttranslational modifications before secretion. These processes included: (a) cleavage of the E‐peptides by the intracellular protease furin convertase to release mature IGF‐1 and E‐peptides, (b) *N*‐glycosylation of the Ea‐peptide, and (c) differential subcellular localization of proIGF‐1s [15, 17]. Because of the complex posttranscriptional and posttranslational regulation of IGF‐1 production, different pools of IGF‐1 proteins might exist intra‐ and extra‐cellularly. Accumulating evidence highlights the distinct roles of IGF‐1 isoforms and Ea‐, Eb‐, or Ec‐peptides in various physiological and pathological contexts. For example, IGF‐1 isoforms have been shown to differentially regulate muscle repair and growth, particularly in response to exercise‐induced muscle damage [19, 20]. They also contribute to cardiac repair following myocardial ischemia and exhibit neuroprotective effects in models of neurodegeneration and ischemia [21]. Furthermore, specific E‐peptides are implicated in modulating inflammation and fibrosis, suggesting isoform‐specific therapeutic potential in conditions such as muscular dystrophy, cardiovascular diseases, and cancer [22, 23]. These findings underscore the necessity for advanced detection systems capable of distinguishing these isoforms, despite the extensive heterogeneity of IGF‐1 proteoforms that complicates their discrimination. Here, to address this challenge, we employed a multiplex fluorescent WB approach using two distinct antibodies, one specific for the IGF‐1 core region and one for the common region of E‐peptides, to simultaneously discriminate the different IGF‐1 isoforms by fluorescence WB.

## 2 Materials and Methods

### 2.1 Cell Transfection and Protein Extraction

Human Embryonic Kidney (HEK) 293 cells were cultured in DMEM at a density of 1.5 × 10^5^ cells per well in a 12‐well plate. HEK293 cells at approximately 60% confluence were transiently transfected using the TransIT‐X2 System (Mirus Bio, LLC, TEMA ricerca) following the manufacturer's instructions. Briefly, the TransIT‐X2:DNA complexes were added to cells in complete growth medium. One microgram of plasmid construct containing the *IGF‐1Ea*, *IGF‐1Eb*, or *IGF‐1Ec* sequence was used. Plasmid details have been previously published [16, 17]. After 24 h of incubation, the HEK293 cells were harvested, centrifuged at 200 × *g* for 5 min and washed once with PBS. Then, HEK293 cell bodies were collected and resuspended in lysis buffer containing: 20 mM HEPES (pH 7.9), 25% v/v glycerol, 0.4 M NaCl, 0.2 mM EDTA, 1.5 mM MgCl_2_, 0.5% v/v Nonidet P‐40, 1.0 mM DTT, 1.0 mM NaF, 1.0 mM Na_3_VO_4_, and 1X complete protease inhibitor cocktail (Roche Diagnostics). The lysates were frozen and thawed twice and clarified by centrifugation at 12 000 × *g* for 10 min at 4°C. The supernatant was collected, and the protein concentration was determined by Bradford assay. Recombinant mature (core) IGF‐1 was purchased from Sigma‐Aldrich (catalog #I3769) while synthetic Ea‐peptide was generously gifted by Dr. Cavalleri (Istituto Nazionale dei Tumori, Milano).

### 2.2 Electrophoresis and WB Analysis

**FIGURE 1:**
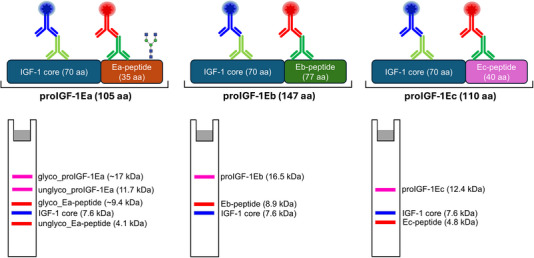
A schematic representation of the experimental protocol of the fluorescent‐based WB and the expected MW of each IGF‐1 isoform. The antibody directed against the core IGF‐1 region and E‐peptides are shown in light green and dark green, respectively. The donkey anti‐rabbit IgG Alexa Fluor Plus 680 and donkey anti‐goat IgG Alexa Fluor Plus 800 secondary fluorescent antibodies are shown in blue and red, respectively. The molecular weight of IGF‐1 isoform was calculated as in Compute pI/Mw (https://web.expasy.org/compute_pi/). Glyco_proIGF‐1Ea: glycosylated proIGF‐1Ea; unglyco_proIGF‐1Ea: unglycosylated proIGF‐1Ea; glyco_Ea‐peptide: glycosylated Ea‐peptide; unglyco_Ea‐peptide: unglycosylated Ea‐peptide.

The protein samples (30 µg total proteins), recombinant mature IGF‐1 protein and synthetic Ea‐peptide were denatured in 2X SDS Laemmli sample buffer with 50 mM dithiothreitol and boiled for 5 min. The samples were separated by SDS‐PAGE (15% acrylamide) in the conventional SDS‐glycine buffer system and transferred to 0.2 µm nitrocellulose membrane (catalog #1620112; Bio‐Rad), or low fluorescence PVDF membrane (catalog #1620264; Bio‐Rad). After transfer, the membranes were blocked with filtered 5% BSA in Tris‐buffered saline (TBS) or with EveryBlot Blocking Buffer (catalog #12010020; Bio‐Rad) at room temperature while shaking for 1 h. After blocking, membranes were incubated overnight at 4°C with a single or a mix of the following primary antibodies resuspended in 5% BSA in TBS‐T (TBS plus 0.05% Tween 20): the rabbit polyclonal anti‐human IGF‐1 antibody which recognizes the core epitope (1:2000 corresponding to 0.125 µg/mL; catalog #500P11; PeproTech) and the goat IGF‐1 polyclonal Ab against E‐peptide domains (1:2000 corresponding to 0.25 µg/mL; catalog #PA5‐19382; Invitrogen). The primary antibody solution was removed, and the membranes were washed with TBS‐T for 15 min for three times. For fluorescence detection, the membranes were incubated in the dark for 1 h at room temperature with TBS‐T containing the mix of the following secondary antibodies: Donkey anti‐rabbit IgG Alexa Fluor Plus 680 (1:20 000, corresponding to 0.10 µg/mL; excitation/emission max 687/704 nm; catalog #A32802; Invitrogen) and donkey anti‐goat IgG Alexa Fluor Plus 800 (1:40 000, corresponding to 0.05 µg/mL; excitation/emission max 789/794 nm; catalog #A32930; Invitrogen). Finally, membranes were washed with TBS‐T in the dark on a shaking platform for 15 min for three times. To remove residual Tween 20, which is highly autofluorescent, the membranes were washed for 5 min in TBS. A schematic representation of the experimental protocol of the fluorescent‐based WB and the expected MW of each IGF‐1 isoform is displayed in Figure 1. For chemiluminescence detection, the immunoreactive bands were revealed by the single appropriate HRP‐conjugated secondary antibodies goat anti‐rabbit IgG (H/L): HRP (1:20 000, catalog #5196‐2504; Bio‐Rad) and rabbit anti‐goat IgG (H/L): HRP (1:5000, catalog #170‐6515; Bio‐Rad). Peroxidase activity was detected with the enhanced chemiluminescence detection method (Clarity ECL Western Blotting Substrates, catalog #1705060; Bio‐Rad). Fluorescencent and chemiluminescent images were obtained on the ChemiDoc MP Imaging System (Bio‐Rad) and analyzed with the Image Lab Touch Software version 6.0.1 (Bio‐Rad) with data analysis, graphing and statistics using GraphPad Prism version 8 (GraphPad Software, Inc., La Jolla, CA, USA).

## 3 Results

**FIGURE 2:**
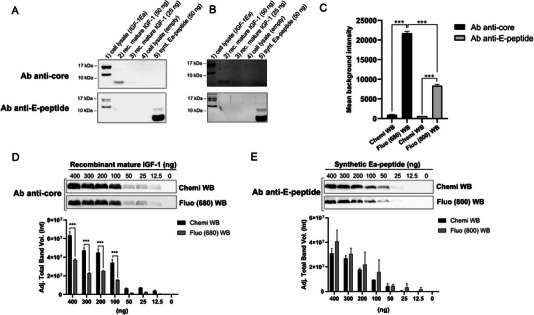
Comparison between WB band patterns obtained by chemiluminescence (A) or fluorescent (B) methods. (C) The background intensity of chemiluminescence and fluorescent blot membranes. (D, E) Bar charts depicting the average signal intensity versus recombinant mature IGF‐1 (D) or synthetic Ea‐peptide (E) obtained by chemiluminescence or fluorescent methods. (1) cell lysate (*IGF‐1Ea*): 30 µg of the cell lysate of HEK293 transiently transfected with a vector containing the human *IGF‐1Ea* sequence, (2) rec. mature IGF‐1 (50 ng): 50 ng of the recombinant mature IGF‐1; (3) rec. mature IGF‐1 (25 ng): 25 ng of the recombinant mature IGF‐1; (4) cell lysate (empty): 30 µg of the cell lysate of HEK293 transiently transfected with an empty vector; (5) synt. Ea‐peptide: 50 ng of the synthetic Ea‐peptide. Ab anti‐core: antibody directed against the core IGF‐1 region; Ab anti‐E‐peptide: antibody directed against the common region of E‐peptides.

**FIGURE 3:**
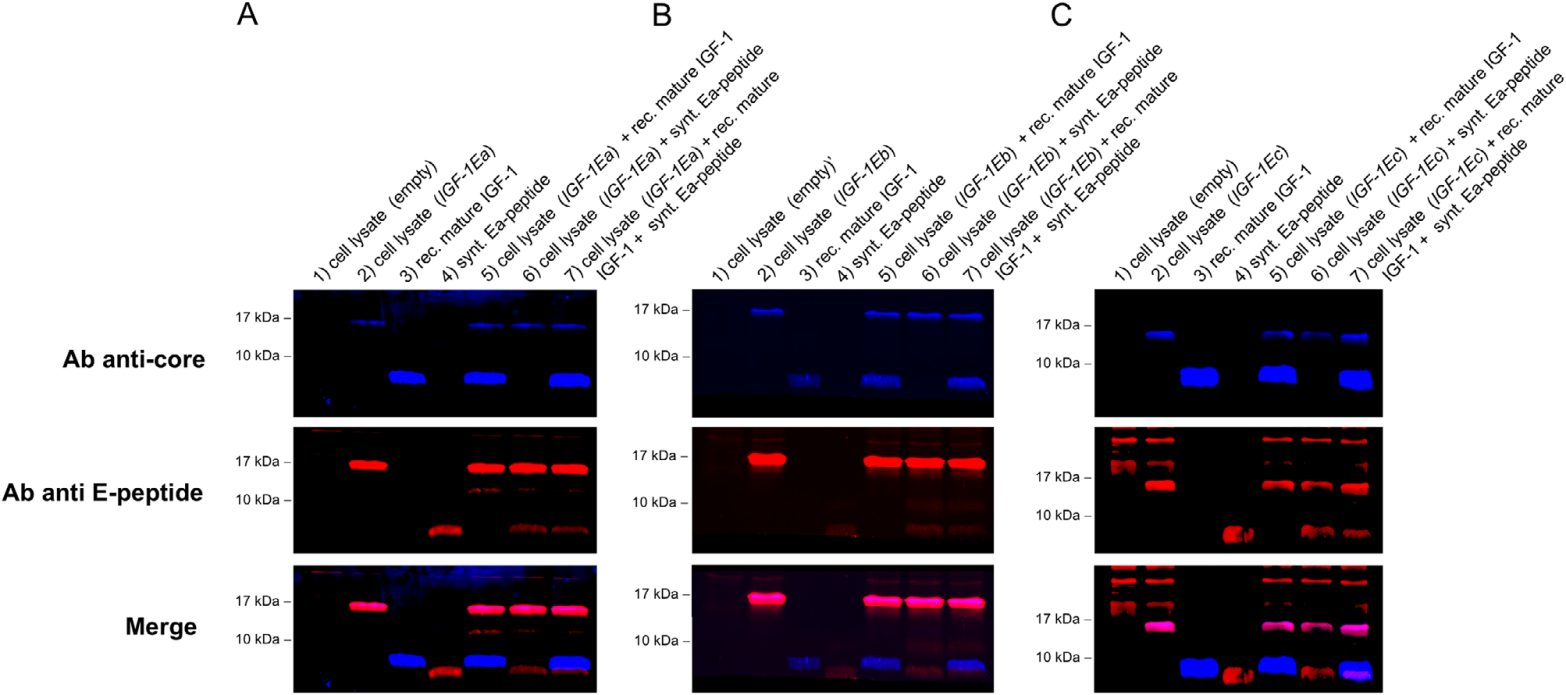
Simultaneously detection of IGF‐1 proteoforms by fluorescent WB. HEK293 cells were transiently transfected with a vector containing the human (A) *IGF‐1Ea*, (B) *IGF‐1Eb*, or (C) *IGF‐1Ec* sequence. (1) cell lysate (empty): 30 µg of the cell lysate of HEK293 transiently transfected with an empty vector; (2) cell lysate (*IGF‐1Ea*, *IGF‐1Eb*, or *IGF‐1Ec*): 30 µg of the cell lysate of HEK293 transiently transfected with a vector containing the human *IGF‐1Ea*, *IGF‐1Eb*, or *IGF‐1Ec* sequence, (3) rec. mature IGF‐1: 50 ng of the recombinant mature IGF‐1 and (4) synt. Ea‐peptide: 50 ng of the synthetic Ea‐peptide. (5) Sample 2 plus 50 ng of the recombinant mature IGF‐1; (6) Sample 2 plus 50 ng of the synthetic Ea‐peptide; (7) Sample 2 plus 50 ng of the recombinant mature IGF‐1 and 50 ng of the synthetic Ea‐peptide. Ab anti‐core: antibody directed against the core IGF‐1 region; Ab anti‐E‐peptide: antibody directed against the common region of E‐peptides.

In an initial experiment, we used a primary antibody targeted against the IGF‐1 core region and another specific to the common region of the E‐peptides to analyze the IGF‐1 pool using a traditional chemiluminescence detection system. The WB analysis presented in Figure 2 shows the protein expression pattern of the lysates of HEK293 cells that were transiently transfected with either a vector containing the human IGF‐1Ea sequence or an empty vector. In addition, the recombinant mature IGF‐1 and the synthetic Ea‐peptide were loaded in WB as positive control. The WB membranes were hybridized with the anti‐core IGF‐1 antibody (Figure 2A upper panel) or the anti‐E‐peptides antibody (Figure 2A, lower panel). Two distinct proteins with a MW of approximately 17 and 12 kDa were detected in the HEK293 cell lysate by the antibody specific to the core IGF‐1 and the E‐peptide. These bands correspond to the glycosylated (expected MW of ∼17 kDa) and unglycosylated proIGF‐1Ea (expected MW of 11.7 kDa) [17]. The recombinant mature IGF‐1 (expected MW of 7.6 kDa) and synthetic Ea‐peptide (expected MW of 4.1 kDa) were recognized only by the anti‐core IGF‐1 antibody and anti‐E‐peptide antibody, as expected. A faint band of approximately 8 kDa was also observed in the synthetic Ea‐peptide sample (Lane 5 of Figure 2A,B) probably representing the Ea‐peptide dimer [17]. Subsequently, we used a similar experimental setting to analyze the IGF‐1 isoforms by a fluorescent WB approach. In particular, a first blot was hybridized with the anti‐core IGF‐1 antibody which was detected by a secondary anti‐rabbit fluorescent antibody (Alexa Fluor Plus 680) (Figure 2B, upper panel) and the second one with the anti‐E‐peptide antibody which was detected by a secondary anti‐goat fluorescent antibody (Alexa Fluor Plus 800) (Figure 2B, lower panel). A marked increase of blot background was found with fluorescent WB compared to chemiluminescence (Figure 2C), especially for the image acquired at 680 nm (anti‐core IGF‐1). We attempted to optimize the signal‐to‐background ratio by using a low‐fluorescent membrane (low fluorescence PVDF membrane; Bio‐Rad Laboratories) and a specific blocking buffer (EveryBlot Blocking Buffer; Bio‐Rad), although none of these strategies were effective to decrease background fluorescence (not shown). Subsequently, we compare the sensitivity between chemiluminescence and fluorescence detection of recombinant mature IGF‐1 (Figure 2D) and synthetic Ea‐peptide (Figure 2E). As anticipated, chemiluminescence detection demonstrated higher sensitivity for the recombinant mature IGF‐1 detection compared to fluorescence detection at 680 nm (Figure 2D). Conversely, the sensitivity of fluorescence detection for the Ea‐peptide (acquired at 800 nm) was comparable to that of chemiluminescence (Figure 2E). However, despite this limitation, the WB banding patterns observed were consistent between the chemiluminescence and fluorescent methods. Afterward, we sought to optimize the simultaneous detection of different IGF‐1 proteoforms by hybridizing the WB membrane with a mixture of the anti‐IGF‐1 core and anti‐E‐peptide antibodies followed by the detection with different fluorescent secondary antibodies. As shown in Figure 3A (upper panel), the anti‐IGF‐1 core antibody recognizes the glycosylated proIGF‐1Ea (expected MW of ∼17 kDa) and the recombinant mature IGF‐1 (expected MW of 7.6 kDa). The anti‐E‐peptide antibody recognizes the glycosylated proIGF‐1Ea (expected MW of ∼17 kDa), the faint band corresponding to unglycosylated proIGF‐1Ea (expected MW of 11.7 kDa) and the recombinant synthetic Ea‐peptide (expected MW of 4.1 kDa) (Figure 3A, middle panel). Notably, merged images of the anti‐IGF‐1 core and the anti‐E‐peptide antibodies allow to show simultaneously the different IGF‐1 proteoforms, easily distinguishing between mature IGF‐1 (blue bands), E‐peptides (red bands) and IGF‐1Ea prohormones (fuchsia bands) (Figure 3A, lower panel). Subsequently, we analyzed HEK293 cells transfected with a vector containing the human *IGF‐1Eb* (Figure 3B) or *IGF‐1Ec* (Figure 3C) sequence. As shown in the merged panel of Figure 3B (lower panel), the expression pattern showed a fuchsia band of approximately 17 kDa representing the *IGF‐1Eb* prohormone (expected MW of 16.5 kDa), and the expected blue and red bands corresponding to the mature IGF‐1 (expected MW of 7.6 kDa) and Ea‐peptide (expected MW of 4.1 kDa), respectively. Similar results were obtained in *IGF‐1Ec* transfected cells (Figure 3C), although several nonspecific bands were observed using the anti‐E‐peptide antibody against the empty‐vector transfected cells. This unexpected result might be due to the low expression level of IGF‐1 proteoforms in the *IGF‐1Ec*‐transfect cells compared to other plasmids.

## 4 Discussion

Here, we describe a strategy for the simultaneous detection of the IGF‐1 proteoforms by fluorescent WB. Using a mixture of two primary antibodies which target distinct epitopes of the IGF‐1 proteoforms, along with two different fluorescently labeled secondary antibodies, we successfully identify and discriminate the different components of the complex IGF‐1 pool in the same blot. Multiplexed detection by fluorescent WB might offer many experimental benefits. Firstly, fluorescent WB avoids stripping and re‐probing or comparing separate blots. These procedures might introduce errors: stripping can cause inconsistent protein loss that compromises quantification; inadequate stripping of antibodies gives rise to spurious or confusing bands; and inconsistencies in loading and transfer cause substantial blot‐to‐blot variation [9]. Second, the ability to unambiguously detect two targets in the same sample lane significantly enhances the accuracy of the analysis, particularly when dealing with proteoforms that have similar MW, such as mature IGF‐1 (expected MW of 7.6 kDa) and Ea‐peptide (expected MW of 4.1 kDa) [10]. The increased experimental throughput and the wide dynamic range of fluorescent WB eliminate the need for the use of long exposure times or ultra‐high sensitivity ECLs, further contributing to its efficiency [7, 9, 10]. Finally, using a mixture of primary antibodies that bind to different epitopes of the same protein some proteoforms were simultaneously detected by the two primary antibodies (e.g. proIGF‐1s). Using merged images these bands were easily identified (e.g. fuchsia bands corresponding to *IGF‐1Ea*, *IGF‐1Eb*, and *IGF‐1Ec* prohormones; Figure 3A–C). The simultaneous detection of the same protein with two different antibodies, typically used in sandwich ELISA, significantly enhances detection specificity. This was dramatically shown by the several nonspecific bands recognized only by the E‐peptide antibody in the negative control sample (HEK293 cells transfected with an empty vector, Figure 3C). None of these bands were also recognized by the anti‐IGF‐1 core antibody (i.e., appeared as fuchsia bands), clearly representing nonspecific antibody binding. The main limit of fluorescent WB remains the membrane autofluorescence, which can only be partially reduced using low‐fluorescent membrane and near‐IR dye‐conjugated secondary antibodies. The higher blot background compared to chemiluminescent can make it challenging to detect and quantify low abundant proteins with fluorescent WB. Despite this, the benefits in terms of specificity, accuracy, and experimental throughput make fluorescent WB a powerful tool for analyzing complex protein samples.

## 5 Concluding Remarks

IGF‐1 is a peptide hormone that promotes the proliferation and differentiation of cells leading to increased growth and development and plays a role in adult tissue maintenance and repair. Synthesized as a precursor protein, IGF‐1 undergoes posttranslational modifications to yield the mature, biologically active form. Given its significant role in various biological processes, the ability to accurately detect and quantify the different isoforms of IGF‐1, including mature IGF‐1, proIGF‐1s, and E‐peptides, within the same sample is highly valuable.

WB is one of the most widely applied and valued methods in biological research, and developing a multiplex fluorescent WB protocol for simultaneous detection and semi‐quantification of IGF‐1 isoforms in a single assay would be particularly beneficial. In particular, such a protocol could be instrumental for mechanistic studies understanding the specific role of IGF‐1 isoforms in cellular processes and signaling pathways, exploring the role of IGF‐1 in cancer biology, where differential expression or modification of IGF‐1 isoforms could contribute to tumorigenesis or cancer progression, and using IGF‐1 and proIGF‐1 as a biomarker in diseases like congenital disorders of glycosylation (CDG), where aberrant proIGF‐1Ea glycosylation has been observed.

In summary, a multiplex fluorescent WB protocol for IGF‐1 isoforms would provide valuable insights into the biological functions and clinical implications of IGF‐1, enhancing our understanding and potential treatment of various diseases.

## Author Contributions

Investigation, visualization, writing–original draft preparation: Matteo Bocconcelli, Fabiana Fanelli and Roberta Saltarelli. Reviewing and editing: Mauro De Santi. Conceptualization, funding acquisition, resources, writing–reviewing and editing: Elena Barbieri, Rita Barone, and Giosuè Annibalini. All the authors contributed to manuscript revision, read and approved the submitted version.

## Conflicts of Interest

The authors declare no conflicts of interest.

## Data Availability

The data that support the findings of this study are available from the corresponding author upon reasonable request.
